# A comparative study of estimators in multilevel linear models

**DOI:** 10.1371/journal.pone.0259960

**Published:** 2021-11-18

**Authors:** Sabz Ali, Said Ali Shah, Seema Zubair, Sundas Hussain

**Affiliations:** 1 Department of Statistics Govt Post Graduate College, Abbottabad, Pakistan; 2 Department of Statistics Govt Post Graduate College, Kohat, Pakistan; 3 Department of statistics, mathematics and computer science, The university of Agriculture, Peshawar, Pakistan; 4 Department of Statistics Shaheed Benazir Bhutto Women University, Peshawar, Pakistan; Tongii University, CHINA

## Abstract

Multilevel Models are widely used in organizational research, educational research, epidemiology, psychology, biology and medical fields. In this paper, we recommend the situations where Bootstrap procedures through Minimum Norm Quadratic Unbiased Estimator (MINQUE) can be extremely handy than that of Restricted Maximum Likelihood (REML) in multilevel level linear regression models. In our simulation study the bootstrap by means of MINQUE is superior to REML in conditions where normality does not hold. Moreover, the real data application also supports our findings in terms of accuracy of estimates and their standard errors.

## Introduction

Multilevel data or clustered data are commonly observed in schools, health institutions, and epidemiology. Multilevel models are also called hierarchical, mixed effects, or random effects models Snijders and Bosker [1], Raudenbush and Bryk [2].

Maximum likelihood (ML) method estimates and estimates standard errors were used by Maas and Hox [3]. Wen et al. [4] concluded that Bayesian spatial-temporal model is superior to the random effects model and spatial model for investigating the effects of weather and roadway characteristics on crash incidence.

Brown and Draper [5] utilized ML method of estimation and accomplished that in small sample sizes the estimates are biased. MINQUE recommended by Rao [6], as an alternate to ML estimator. The method, however, does not rely on the assumption of normality in multilevel linear models. According to Bagakas [7], one major problem with the MINQUE estimators is that standard errors of the minimum norm quadratic unbiased estimators cannot be computed because of the non-existence of formulae. In situations, where a researcher attempts to construct confidence interval and perform testing of hypothesis about the parameter then the MINQUE is not appropriate. The researcher then needs to use an alternate scheme such as bootstrapping, where not only the parameter estimates but also their standard errors can be estimated by applying different estimation methods such as MINQUE or ML method of estimation.

In practice, both parametric and nonparametric bootstrap can be used. However, when the assumption of normality does not exist the nonparametric bootstrap is handy. As the MINQUE method of estimation is free from the normality assumption, so the bootstrap by means of MINQUE will be used. Swallow and Monahan [8] compared REML, ML and MINQUE estimators.

Bagakas [7] used bootstrap by means of MINQUE. Similarly, Meijer et al. [9] concluded that multilevel bootstrapping performance was excellent in small sample sizes in multilevel models. Carpenter et al. [10] carried out a simulation study where they compared the relative performance of parametric bootstrap and nonparametric residuals bootstrap methods by using multilevel linear models. Hutchison et al. [11] successfully carried out simulation study on a two-level model. They applied the procedure of nonparametric cases bootstrap and promising standard errors of the estimates were obtained. Wang et al. [12] used multilevel linear model to apply nonparametric residual bootstrap through a SAS macro. Nonparametric residual bootstrap estimates standard errors were promising. Delpish [13] also compared REML and Bootstrap by means of MINQUE in her study. Ali et al., [14] concluded that ML gave better results than Penalized Quasilikelihood (PQL)for small sample conditions in multilevel model. To get accurate estimates of both fixed and random effects ML requires relatively small sample compared to PQL in multilevel logistic models (Ali et al. [15]). In a study by Zeng et al. [16] revealed that univariate spatial model gave lower deviance information criteria (DIC) and accurate estimates of parameters as compared to bivariate spatial model while investigating the factors responsible for vehicle crash on freeway. The proposed multivariate random-parameters spatio-temporal Tobit model gave lower Deviance Information Criteria (DIC), Mean Absolute Deviance (MAD) and Mean Squared Prediction Errors (MSPE) then the competing model such as multivariate random-parameters Tobit model and a multivariate random-parameters spatial Tobit model (Zeng et al. [17]. It was confirmed from the results that spatio-temporal correlation and interaction have significance in the area wide crash data.

In this paper, the researchers investigate the performance of REML and Bootstrap by means of MINQUE under varying conditions of the number of groups, Intra-class correlation and different skewed distributions.

## Materials and methods

For this study a random intercept and random slope multilevel linear model was used. The model has single explanatory variable at each level. The model is given below:

$$Y_{ij}=\beta_{0j}+\beta_{1j}X_{ij}+e_{ij}$$ (1)

Level 1 model

$$\beta_{0j}=\gamma_{00}+\gamma_{01}W_{j}+u_{oj}$$ (2)

Level 2 models

$$\beta_{1j}=\gamma_{10}+\gamma_{11}W_{j}+u_{1j}$$ (3)

The combined model was obtained by substituting level 2 model in level 1 model:

$$Y_{ij}=(\gamma_{00}+\gamma_{10}X_{ij}+\gamma_{01}W_{j}+\gamma_{11}X_{ij}W_{j})+(u_{oj}+u_{1j}X_{ij}+e_{ij})$$ (4)

(Fixed part)+(Random part)

Where *X*_*ij*_ is the Level 1 explanatory variable, *W*_*j*_ corresponds to Level 2 explanatory variable, *γ*_00_, *γ*_10_, *γ*_01_ and *γ*_11_ are the fixed effects, *e*_*ij*_ is assumed to follows a normal distribution i.e

*e*_*ij*_∼ N (0, $\sigma_{e}^{2}$). In case of normality, *u*_*oj*_ and *u*_1*j*_ assumed to follow a multivariate normal distribution as

$$\left[\begin{array}{c} u_{oj}\\ u_{1j} \end{array}\right]∼N\left([\begin{array}{c} 0\\ 0 \end{array}],[\begin{array}{cc} \sigma_{u}^{2} & \sigma_{u1}\\ \sigma_{u1} & \sigma_{1}^{2} \end{array}]\right)$$ (5)

$\sigma_{u}^{2}$ Corresponds to the random intercept variance, $\sigma_{1}^{2}$ is the random slope variance and *σ*_*u*1_ is the covariance term.

### Design factors

1. Three levels of number of groups were used in this study: 30,100 and 120.
2. Three levels of intra-class correlations were used: 0.01, 0.10 and 0.20. Where the intra-class correlation coefficient (ICC) is given as

$$ICC=\frac{\sigma_{u}^{2}}{\sigma_{u}^{2}+\sigma_{e}^{2}}$$ (6)

3. Three distributions were used: Normal distribution, Lognormal distribution and Exponential distribution

### Analysis

Two estimation procedures Restricted Maximum Likelihood and Bootstrap by means of MINQUE were used in all the three distribution conditions. All the simulations and bootstrapping were performed in SAS 9.2 to obtain estimates and their standard errors.

**Algorithm.** The procedure for cases bootstrap is as given below:

1. Draw with replacement *J* group level units along with corresponding scores on group level variable $W_{j}^{*}$.
2. Then draw with replacement *n*_*j*_ individual level units within group level unit *j*, *j* = 1, 2………, *J*. This results the bootstrap data (Y*, X*) and this data set is then combined with the group level variable $W_{j}^{*}$ in order to get (Y*, X*, $W_{j}^{*}$) the desired bootstrap sample.
3. Obtain the minimum norm quadratic unbiased estimates of the model parameters from the bootstrap replicated sample.
4. Replicate steps 1–3 B times, b = 1, 2, 3…… B, and then obtain the minimum norm quadratic unbiased estimates of the model parameters.
5. Obtain the mean value of estimates by using

$${\hat{\theta }}^{*}(.)=\frac{1}{B}\overset{B}{\underset{b=1}{\sum }}{\hat{\theta }}^{*}(b)$$ (7)

And the bootstrap parameter estimate standard error is obtain as

$$s{.e}_{B}=[\overset{B}{\underset{b=1}{\sum }}\frac{{\left({\hat{\theta }}^{*}(b)-{\hat{\theta }}^{*}(.)\right)}^{2}}{B-1}){]}^{1/2}$$ (8)

The real data was selected from High School & Beyond Survey data set, which is a national survey of United States conducted by National Center for Educations Statistics (NCES) about Public and Catholic schools. For the purpose of illustration, a dataset of 30 schools was randomly selected from the data of 160 schools.

## Results

Tables 1 and 2 show that the bootstrap procedure showed perfect results in terms of accuracy of the fixed and random effects estimates, however, REML method estimates were comparable to that of the bootstrap procedure at 100 and 1200 groups respectively. Similarly, from Table 3 it is evident that the bootstrap CI outclassed the REML CI at the first two levels of the number of group (30 and 100) factor when the distribution was normal.

**Table 1:** Average relative parameter bias of fixed effects estimates obtained for normal distribution data (First = REML estimation procedure, Second = Bootstrap estimates are enclosed in parenthesis).

Groups	ICC	*γ*_00_	*γ*_10_	*γ*_01_	*γ*_11_
30	0.01	-0.0209**/ (**0.0037)	-0.0182**/ (**0.0028)	-0.0149**/ (**0.0019)	0.0164**/ (**0.0031)
	0.10	-0.0311**/ (**0.0041)	-0.0244**/ (**0.0029)	-0.0206**/ (**0.0022)	0.0196**/ (**0.0036)
	0.20	-0.0389**/ (**0.0055)	-0.0401**/ (**0.0046)	-0.0315**/ (**0.0022)	0.0283**/ (**0.0041)
100	0.01	0.0051**/ (**0.0000)	0.0029**/ (**0.0000)	-0.0039**/ (**0.0002)	0.0018**/ (**0.0004)
	0.10	-0.0096**/ (**0.0009)	0.0069**/ (**0.0004)	0.0032**/ (**0.0008)	0.0055**/ (**0.0009)
	0.20	-0.0126**/ (**0.0024)	-0.0093**/ (**0.0014)	0.0088**/ (**0.0010)	0.0079**/ (**0.0012)
120	0.01	0.0004**/ (**0.0000)	0.0002**/ (**0.0000)	-0.0006**/ (**0.0000)	0.0000**/ (**0.0000)
	0.10	0.0009**/ (**0.0001)	0.0012**/ (**0.0000)	0.0016**/ (**0.0000)	0.0010**/ (**0.0000)
	0.2	0.0013**/ (**0.0002)	0.0018**/ (**0.0001)	0.0021**/ (**0.0000)	0.0015**/ (**0.0000)

**Table 2:** Average relative parameter bias of the random effects estimates obtained for normal distribution data (First = REML estimation procedure, Second = Bootstrap estimates are enclosed in Parenthesis).

Groups	ICC	*σ*_*u*_	*σ*_1_	*σ*_*u*1_	*σ*_*e*_
30	0.01	-0.0411**/ (**0.0082)	-0.0366**/ (**0.0049)	-0.0249**/ (**0.0073)	-0.0039**/ (**0.0003)
	0.10	-0.0544**/ (**0.0121)	-0.0485**/ (**0.0097)	-0.0402**/ (**0.0081)	-0.0053**/ (**0.0011)
	0.20	-0.0719**/ (**0.0129)	-0.0679**/ (**0.0141)	-0.0593**/ (**0.0109)	-0.0066**/ (**0.0020)
100	0.01	-0.0207**/ (**0.0039)	-0.0216**/ (**0.0019)	-0.0172**/ (**0.0026)	-0.0009**/ (**0.0000)
	0.10	-0.0231**/ (**0.0051)	-0.0238**/ (**0.0033)	-0.0189**/ (**0.0046)	-0.0013**/ (**0.0000)
	0.20	-0.0301**/ (**0.0057)	-0.0251**/ (**0.0046)	-0.0201**/ (**0.0053)	-0.0016**/ (**0.0001)
120	0.01	-0.0060**/ (**0.0000)	-0.0048**/ (**0.0000)	-0.0019**/ (**0.0001)	0.0000**/ (**0.0000)
	0.10	-0.0088**/ (**0.0001)	-0.0079**/ (**0.0000)	-0.0032**/ (**0.0002)	0.0000**/ (**0.0000)
	0.2	-0.0101**/ (**0.0002)	-0.0089**/ (**0.0000)	-0.0061**/ (**0.0002)	0.0000**/ (**0.0000)

**Table 3:** Impact of groups and ICC on estimates 95% coverage probability for normal distribution data (First = REML estimation procedure, Second = Percentile bootstrap estimates are enclosed in Parenthesis).

Parameters	Groups			P-value	ICC			P-value
	30	100	120		0.01	0.10	0.20	
*γ*_00_	0.935 **(**0.940)	0.947 **(**0.952)	0.961 **(**0.962)	0.00 (0.00)	0.948 (0.949)	0.948 (0.951)	0.947 (0.953)	0.77 (0.51)
*γ*_10_	0.940 (0.950)	0.949 (0.959)	0.962 (0.965)	0.00 (0.00)	0.950 (0.959)	0.949 (0.956)	0.950 (0.956)	0.95 (0.60)
*γ*_01_	0.939 (0.951)	0.948 (0.956)	0.958 (0.960)	0.00 (0.07)	0.948 (0.958)	0.950 (0.955)	0.948 (0.953)	0.90 (0.31)
*γ*_11_	0.939 (0.948)	0.951 (0.955)	0.960 (0.960)	0.00 (0.03)	0.951 (0.956)	0.951 (0.955)	0.948 (0.952)	0.72 (0.53)
*σ*_*u*_	0.907 (0.946)	0.935 (0.950)	0.946 (0.950)	0.00 (0.21)	0.928 (0.951)	0.930 (0.949)	0.933 (0.948)	0.44 (0.63)
*σ*_1_	0.906 (0.948)	0.929 (0.950)	0.946 (0.950)	0.00 (0.76)	0.924 (0.950)	0.927 (0.948)	0.930 (0.948)	0.32 (0.76)
*σ*_*u*1_	0.914 (0.950)	0.930 (0.955)	0.952 (0.956)	0.00 (0.29)	0.930 (0.955)	0.932 (0.952)	0.933 (0.952)	0.68 (0.71)
*σ*_*e*_	0.945 (0.949)	0.950 (0.953)	0.954 (0.955)	0.09 (0.25)	0.951 (0.954)	0.948 (0.953)	0.950 (0.949)	0.90 (0.36)

The bootstrap procedure was superior to REML in terms of accuracy of the fixed effects and random effect estimates as can be seen in Tables 4 and 5 for lognormal distribution. Moreover, Table 6 reveals that the bootstrap CI outperformed the REML CI at all levels of the number of groups when data was generated from lognormal distribution. Furthermore, when the distribution of the data was exponential again the bootstrap method outshined the REML method estimates as shown in Tables 7–9 respectively.

**Table 4:** Average relative parameter bias of fixed effect estimates obtained for lognormal distribution data (First = REML estimation procedure, Second = Bootstrap estimates are enclosed in parenthesis).

Groups	ICC	*γ*_00_	*γ*_10_	*γ*_01_	*γ*_11_
30	0.01	-0.0169**/ (**0.0015)	0.0148**/ (**0.0010)	0.0171**/ (**0.0008)	-0.0200**/ (**0.0012)
	0.10	-0.0315**/ (**0.0016)	0.0211**/ (**0.0030)	0.0219**/ (**0.0011)	-0.0291**/ (**0.0015)
	0.20	-0.0409**/ (**0.0019)	0.0397**/ (**0.0050)	0.0412**/ (**0.0020)	-0.0329**/ (**0.0019)
100	0.01	0.0039**/ (**0.0000)	0.0081**/ (**0.0000)	0.0066**/ (**0.0000)	0.0055**/ (**0.0004)
	0.10	-0.0051**/ (**0.0002)	0.0097**/ (**0.0000)	0.0073**/ (**0.0003)	-0.0069**/ (**0.0009)
	0.20	-0.0056**/ (**0.0006)	0.0110**/ (**0.0002)	0.0081**/ (**0.0003)	-0.0089**/ (**0.0012)
120	0.01	0.0002**/ (**0.0000)	0.0000**/ (**0.0000)	0.0000**/ (**0.0000)	0.0001**/ (**0.0000)
	0.10	0.0006**/ (**0.0000)	0.0001**/ (**0.0000)	0.0006**/ (**0.0000)	0.0011**/ (**0.0000)
	0.2	0.0009**/ (**0.0000)	0.0004**/ (**0.0000)	0.0012**/ (**0.0000)	0.0015**/ (**0.0000)

**Table 5:** Average relative parameter bias of the random effect estimates obtained for lognormal distribution data (First = REML estimation procedure, Second = Bootstrap estimates are enclosed in parenthesis).

Groups	ICC	*σ*_*u*_	*σ*_1_	*σ*_*u*1_	*σ*_*e*_
30	0.01	-0.0398**/ (**0.0102)	-0.0501**/ (**0.0129)	-0.0289**/ (**0.0029)	-0.0091**/ (**0.0002)
	0.10	-0.0611**/ (**0.0137)	-0.0721**/ (**0.0148)	-0.0377**/ (**0.0041)	-0.0122**/ (**0.0002)
	0.20	-0.0814**/ (**0.0152)	-0.0912**/ (**0.0162)	-0.0602**/ (**0.0066)	-0.0147**/ (**0.0003)
100	0.01	-0.0194**/ (**0.0018)	-0.0279**/ (**0.0021)	-0.0251**/ (**0.0018)	0.0011**/ (**0.0000)
	0.10	-0.0206**/**0.0024)	-0.0296**/ (**0.0023)	-0.0302**/ (**0.0021)	0.0015**/ (**0.0001)
	0.20	-0.0213**/ (**0.0027)	-0.0310**/ (**0.0026)	-0.0320**/ (**0.0026)	0.0015**/ (**0.0002)
120	0.01	-0.0085**/ (**0.0000)	-0.0092**/ (**0.0000)	-0.0059**/ (**0.0000)	-0.0003**/ (**0.0000)
	0.10	-0.0095**/ (**0.0000)	-0.0096**/ (**0.0000)	-0.0065**/ (**0.0000)	-0.0005**/ (**0.0000)
	0.2	-0.0103**/ (**0.0002)	-0.0097**/ (**0.0001)	-0.0070**/ (**0.0000)	-0.0006**/ (**0.0000)

**Table 6:** Impact of groups ICC on estimates 95% coverage probability for lognormal distribution data (First = REML estimation procedure, Second = Percentile bootstrap estimates are enclosed in parenthesis).

Parameters	Groups			P-value	ICC			P-value
	30	100	120		0.01	0.10	0.20	
*γ*_00_	0.914 **(**0.944)	0.930 **(**0.949)	0.948 **(**0.952)	0.00 (0.20)	0.936 (0.952)	0.931 (0.948)	0.925 (0.945)	0.12 (0.18)
*γ*_10_	0.921 (0.947)	0.936 (0.952)	0.944 (0.954)	0.00 (0.18)	0.936 (0.955)	0.934 (0.952)	0.930 (0.947)	0.36 (0.13)
*γ*_01_	0.918 (0.945)	0.929 (0.951)	0.945 (0.955)	0.00 (0.21)	0.935 (0.953)	0.931 (0.951)	0.926 (0.946)	0.18 (0.21)
*γ*_11_	0.916 (0.941)	0.930 (0.947)	0.945 (0.950)	0.00 (0.15)	0.934 (0.951)	0.929 (0.946)	0.928 (0.941)	0.31 (0.09)
*σ*_*u*_	0.702 (0.898)	0.734 (0.905)	0.759 (0.908)	0.00 (0.22)	0.752 (0.909)	0.732 (0.904)	0.712 (0.898)	0.00 (0.17)
*σ*_1_	0.709 (0.906)	0.731 (0.911)	0.766 (0.915)	0.00 (0.20)	0.756 (0.915)	0.732 (0.910)	0.716 (0.907)	0.00 (0.28)
*σ*_*u*1_	0.722 (0.915)	0.749 (0.919)	0.779 (0.921)	0.00 (0.37)	0.760 (0.921)	0.749 (0.919)	0.741 (0.915)	0.10 (0.38)
*σ*_*e*_	0.920 (0.944)	0.931 (0.948)	0.935 (0.950)	0.02 (0.24)	0.930 (0.950)	0.928 (0.947)	0.928 (0.945)	0.76 (0.34)

**Table 7:** Average relative parameter bias of fixed effect estimates obtained for exponential distribution data (First = REML estimation procedure, Second = Bootstrap estimates are enclosed in parenthesis).

Groups	ICC	*γ*_00_	*γ*_10_	*γ*_01_	*γ*_11_
30	0.01	0.0221**/ (**0.0022)	-0.0237**/ (**0.0006)	0.02456 **(**0.0013)	0.0207**/ (**0.0007)
	0.10	0.0344**/ (**0.0024)	-0.0401**/ (**0.0012)	0.0364**/ (**0.0016)	0.0418**/ (**0.0010)
	0.20	0.0424**/ (**0.0029)	-0.0515**/ (**0.0015)	0.0479**/ (**0.0019)	0.0461**/ (**0.0013)
100	0.01	0.0089**/ (**0.0001)	0.0116**/ (**0.0000)	0.0082**/ (**0.0001)	0.0049**/ (**0.0000)
	0.10	0.0103**/ (**0.0002)	-0.0129**/ (**0.0001)	0.0089**/ (**0.0002)	-0.0057**/ (**0.0001)
	0.20	0.0111**/ (**0.0002)	-0.0141**/ (**0.0003)	0.0093**/ (**0.0003)	-0.0059**/ (**0.0002)
120	0.01	0.0016**/ (**0.0000)	0.0030**/ (**0.0000)	0.0008**/ (**0.0000)	0.0005**/ (**0.0000)
	0.10	0.0022**/ (**0.0000)	-0.0033**/ (**0.0000)	0.0013**/ (**0.0000)	0.0016**/ (**0.0000)
	0.20	0.0029**/ (**0.0000)	-0.0039**/ (**0.0000)	0.0015**/ (**0.0000)	0.0016**/ (**0.0000)

**Table 8:** Average relative parameter bias of the random effect estimates obtained for exponential distribution data (First = REML estimation procedure, Second = Bootstrap estimates are enclosed in parenthesis).

Groups	ICC	*σ*_*u*_	*σ*_1_	*σ*_*u*1_	*σ*_*e*_
30	0.01	-0.0359**/ (**0.0122)	-0.0446**/ (**0.0164)	-0.0377**/ (**0.0101)	-0.0114**/ (**0.0004)
	0.10	-0.0572**/ (**0.0149)	-0.0634**/ (**0.0164)	-0.0495**/ (**0.0104)	-0.0140**/ (**0.0005)
	0.20	-0.0786**/ (**0.0182)	-0.0809**/ (**0.0172)	-0.0584**/ (**0.0114)	-0.0161**/ (**0.0006)
100	0.01	-0.0224**/ (**0.0022)	-0.0251**/ (**0.0028)	-0.0277**/ (**0.0045)	0.0025**/ (**0.0000)
	0.10	-0.0239**/ (**0.0028)	-0.0269**/ (**0.0039)	-0.0297**/ (**0.0049)	-0.0029**/ (**0.0003)
	0.20	-0.0270**/ (**0.0031)	-0.0280**/ (**0.0051)	-0.0306**/ (**0.0049)	-0.0032**/ (**0.0003)
120	0.01	-0.0102**/ (**0.0000)	-0.0081**/ (**0.0000)	-0.0088**/ (**0.0000)	0.0008**/ (**0.0000)
	0.10	-0.0106**/ (**0.0000)	0.0091**/ (**0.0000)	-0.0089**/ (**0.0001)	0.0008**/ (**0.0000)
	0.2	-0.0109**/ (**0.0001)	-0.0102**/ (**0.0002)	-0.0094**/ (**0.0001)	0.0011**/ (**0.0001)

**Table 9:** Impact of groups and ICC on estimates 95% coverage probability for exponential distribution data (First = REML estimation procedure, Second = Percentile bootstrap estimates are enclosed in parenthesis).

Parameters	Groups			P-value	ICC			P-value
	30	100	120		0.01	0.10	0.20	
*γ*_00_	0.914 **(**0.944)	0.930 **(**0.949)	0.948 **(**0.952)	0.00 (0.20)	0.943 (0.956)	0.939 (0.953)	0.934 (0.949)	0.11 (0.24)
*γ*_10_	0.921 (0.947)	0.936 (0.952)	0.944 (0.954)	0.00 (0.18)	0.936 (0.957)	0.931 (0.953)	0.927 (0.948)	0.15 (0.11)
*γ*_01_	0.918 (0.945)	0.929 (0.951)	0.945 (0.955)	0.00 (0.21)	0.947 (0.954)	0.942 (0.950)	0.939 (0.946)	0.20 (0.14)
*γ*_11_	0.916 (0.941)	0.930 (0.947)	0.945 (0.950)	0.00 (0.15)	0.941 (0.949)	0.936 (0.946)	0.931 (0.941)	0.12 (0.17)
*σ*_*u*_	0.702 (0.898)	0.734 (0.905)	0.759 (0.908)	0.00 (0.22)	0.762 (0.948)	0.735 (0.944)	0.701 (0.940)	0.00 (0.21)
*σ*_1_	0.709 (0.906)	0.731 (0.911)	0.766 (0.915)	0.00 (0.20)	0.749 (0.945)	0.722 (0.942)	0.695 (0.940)	0.00 (0.31)
*σ*_*u*1_	0.722 (0.915)	0.749 (0.919)	0.779 (0.921)	0.00 (0.37)	0.763 (0.943)	0.739 (0.940)	0.711 (0.936)	0.10 (0.23)
*σ*_*e*_	0.920 (0.944)	0.931 (0.948)	0.935 (0.950)	0.02 (0.24)	0.945 (0.951)	0.939 (0.948)	0.933 (0.945)	0.06 (0.32)

### Real data application

The application of bootstrap by means of MINQUE method to the real data is demonstrated in this section. A two-level model was fitted to a subsample data drawn from High School & Beyond (HSB) data. The data consist of two levels i.e school level and student level. HSB data consists of 7185 students nested within 160 schools. The data contains four level 1 or individual level variables and six level 2 or group level variables in total. For the purpose of illustration of bootstrap by means of MINQUE method only 30 schools were drawn randomly from 160 schools. The total numbers of level 1 units are 1447 and level 2 units are 30. Students MATH ACHIEVEMENT SCORE was taken as a response variable, SES was selected as a level 1 variable and MEANSES was selected as a level 2 variable. A two-level model used in this real data application is given below

$$Y_{ij}=\beta_{0j}+\beta_{1j}SES+e_{ij}$$ (9)

Level-1 model

$$\beta_{0j}=\gamma_{00}+\gamma_{01}MEANSES+u_{oj}$$ (10)

Level-2 models

$$\beta_{1j}=\gamma_{10}+\gamma_{11}MEANSES+u_{1j}$$

$$i=1,2,\ldots n_{j}\;\mathrm{and}\;j=1,2,\ldots J$$

The combined model can be written as

$$Y_{ij}=(\gamma_{00}+\gamma_{10}{SES}_{ij}+\gamma_{01}{MEANSES}_{j}+\gamma_{11}({SES}_{ij})({MEANSES}_{j}))+(u_{oj}+u_{1j}{SES}_{ij}+e_{ij)}$$ (11)

REML and bootstrap by means of MINQUE estimation procedures were used to estimate both fixed effects and random effects using HSB: 30 schools data set for the model in equation (1.8). The SAS package procedure PROC MIXED was used to obtain REML estimates and estimates standard errors. The REML confidence intervals were then constructed for each parameter using normal theory. For all the eight parameters in the model (1.8), B = 1000 bootstrap replicates were obtained using cases bootstrap. The mean of 1000 estimates were then taken to obtain the bootstrap estimate. This means that the bootstrap estimate of any parameter is the average of one thousand estimates. On the other hand, single estimate for each parameter was obtained under REML method of estimation. Bootstrap confidence intervals were constructed for each parameter in the model by using the percentile method. The data set of 30 schools randomly selected from 160 school’s data is presented in Table 10.

**Table 10:** High school & beyond data (30 schools data set).

Serial number	School ID	Number of level 1 units per school	Serial number	School ID	Number of level 1 units per school
1	1224	47	16	4458	48
2	1308	20	17	4868	34
3	1358	30	18	5192	28
4	1433	35	19	5650	45
5	1477	62	20	5762	37
6	2277	61	21	5783	29
7	2467	52	22	5838	31
8	2771	55	23	6074	56
9	3039	21	24	6144	43
10	3332	38	25	6291	35
11	3610	64	26	6443	30
12	4223	45	27	6464	29
13	4325	53	28	6484	35
14	4350	33	29	6600	56
15	4410	41	30	7688	54

Total number of level 2 units = 30 schools, Total number of level 1 units = 1447 students.

Table 11 illustrates estimates and estimated standard errors under REML and bootstrap by means of MINQUE methods of estimation. Moreover, 95% CI’s are also given in Table 11. There is not much difference to choose between the two procedures as for as the accuracy of the estimates is concerned. However, both fixed and random effects estimate standard errors were lower under bootstrap by means of MINQUE. The widths of the REML CI’s were clearly higher than that of the percentile bootstrap CI’s. Overall, for real data, bootstrap by means of MINQUE performs better than that of the REML method of estimation especially in terms of precision. Simulation results also exposed that bootstrap by means of MINQUE procedure outperformed the REML method of estimation particularly in terms of estimates promising standard errors.

**Table 11:** Fixed and random effects parameter estimates and CI Limits under both REML and bootstrap by means of MINQUE methods of estimation for real data.

Parameters	Estimation Method	Estimate	S.Error	L.L	U.L	Interval Width
*γ*_00_	REML	12.9118	0.3366	12.2521	13.5716	1.3195
	Bootstrap	12.8811	0.2413	12.8383	13.4342	0.5959
*γ*_10_	REML	2.0665	0.3095	1.4599	2.6731	1.2132
	Bootstrap	2.0641	0.1710	1.7690	2.4292	0.6602
*γ*_01_	REML	5.0705	0.7971	3.5081	6.6329	3.1248
	Bootstrap	5.0301	0.7004	3.6674	6.4228	2.7554
*γ*_11_	REML	0.7676	0.6544	-0.5150	2.0502	2.5652
	Bootstrap	0.7478	0.5224	-0.2986	1.8037	2.1023
*σ*_*u*_	REML	1.4151	0.2721	0.8817	1.9485	1.0668
	Bootstrap	1.4202	0.2024	1.1335	1.9169	0.7834
*σ*_1_	REML	0.7216	0.3929	-0.0485	1.4917	1.5367
	Bootstrap	0.7215	0.2749	0.2127	1.2953	1.0826
*σ*_*u*1_	REML	-0.0598	0.5034	-1.0465	0.9269	1.9734
	Bootstrap	-0.6220	0.4121	-1.3597	0.2457	1.6054
*σ*_*e*_	REML	6.0399	0.1235	5.7978	6.2820	0.4842
	Bootstrap	6.0399	0.0666	5.8994	6.1504	0.2510

## Conclusion

REML produced unbiased fixed effects estimates at the second level and third level of the number of groups (100 and 120) factor. On the other hand, the bootstrap fixed effects estimates were unbiased across all conditions. Additionally, the bootstrap procedure outperformed the REML method in terms of accuracy of the random effects estimates when the number of groups was 30. Based on the above normal data results, it is recommended that at least 30 groups are essential to obtain unbiased fixed effects estimates and their standard errors under REML method of estimation. Furthermore, 100 groups are essential to achieve accurate random effects estimates and their standard errors under REML method of estimation. It is also recommended that bootstrap by means of MINQUE can be superior to REML when the number of groups are 30 and normality holds.

In general, the estimates and estimated standard errors were biased for the two skewed distribution data when the number of groups was 30 under REML method of estimation. On the other hand, the bootstrap estimates and estimated standard errors were unbiased across all conditions. To put it differently, the bootstrap fixed effects and random effects estimates coverage rates were not only acceptable but also superior to that of REML estimates coverage rates across all conditions. Furthermore, REML level 2 random effects estimates coverage rates were unacceptable across all conditions under both skewed distributions data. Moreover, real data results and conclusion are clearly matching with the simulation results.

It is recommended on the basis of these study results, whenever the data are based on skewed distributions and normality assumption does not hold, REML should not be used particularly for inference. In such situations, the bootstrap standard errors by means of MINQUE can be used for inference to achieve precise results.

## Supporting information

S1 File (RAR)
